# Patterns of Dendritic Basal Field Orientation of Pyramidal Neurons in the Rat Somatosensory Cortex

**DOI:** 10.1523/ENEURO.0142-18.2018

**Published:** 2019-01-17

**Authors:** Ignacio Leguey, Ruth Benavides-Piccione, Concepción Rojo, Pedro Larrañaga, Concha Bielza, Javier DeFelipe

**Affiliations:** 1 Departamento de Economía Financiera y Contabilidad e Idioma Moderno, Facultad de Ciencias Jurídicas y Sociales, Universidad Rey Juan Carlos de Madrid, Vicálvaro 28042, Spain; 2 Departamento de Inteligencia Artificial, Escuela Técnica Superior de Ingenieros Informáticos, Universidad Politécnica de Madrid, Boadilla del Monte 28660, Spain; 3 Laboratorio Cajal de Circuitos Corticales, Centro de Tecnología Biomédica, Universidad Politécnica de Madrid, Pozuelo de Alarcón 28223, Spain; 4 Consejo Superior de Investigaciones Científicas, Instituto Cajal, Madrid 28040, Spain

**Keywords:** pyramidal cell morphology, circular statistics, neuron orientation

## Abstract

The study of neuronal dendritic orientation is of interest because it is related to how neurons grow dendrites to establish the synaptic input that neurons receive. The dendritic orientations of neurons in the nervous system vary, ranging from rather heterogeneously distributed (asymmetric) to homogeneously distributed (symmetric) dendritic arbors. Here, we analyze the dendritic orientation of the basal dendrites of intracellularly labeled pyramidal neurons from horizontal sections of Layers II–VI of the hindlimb somatosensory (S1HL) cortex of 14-d-old (P14) rats. We used circular statistics and proposed two new graphical descriptive representations of the neuron. We found that the dendritic pattern of most neurons was asymmetric. Furthermore, we found that there is a mixture of different types of orientations within any given group of neurons in any cortical layer. In addition, we investigated whether dendritic orientation was related to the physical location within the brain with respect to the anterior, dorsal, posterior and ventral directions. Generally, there was a preference towards the anterior orientation. A comparison between layers revealed that the preference for the anterior orientation was more pronounced in neurons located in Layers II, III, IV, and Va than for the neurons located in Layers Vb and VI. The dorsal orientation was the least preferred orientation in all layers, except for Layers IV and Va, where the ventral orientation had the lowest preference. Therefore, the orientation of basal dendritic arbors of pyramidal cells is variable and asymmetric, although a majority has a single orientation with a preference for the anterior direction in P14 rats.

## Significance Statement

It is thought that the dendritic arborizations of pyramidal cells are oriented to make contact with relevant synaptic inputs. It could be expected that these arborizations are distributed uniformly around the soma. We used circular statistics and two new graphical descriptive representations of the neuron to study the orientation of basal dendritic arbors in the rat somatosensory cortex. We observed that the basal dendritic pattern of most neurons was asymmetric and that was a mixture of different types of orientations within any given group of neurons in any cortical layer. Nevertheless, we found a large proportion of cells showing a preference for the anterior orientation axis, suggesting that these cells have a preference for an axonal system oriented in this direction.

## Introduction

Pyramidal cells are the most abundant neuronal type in the cerebral cortex. Their basic microanatomical structure is well characterized and rather stereotyped: there are two main dendritic arbors, the apical and the basal arbors. Apical dendrites emerge from the upper pole of the soma as a prominent process directed radially toward the pia, giving off a number of thinner oblique branches during their ascending trajectory. The basal dendrites emerge from the base of the soma and are directed sidewards or downwards. It is well established that these two main dendritic arbors have different morphologic and functional features and are involved in different synaptic circuits (DeFelipe and Fariñas, 1992; Spruston, 2008; Larkum, 2013; Harris and Shepherd, 2015; Mel et al., 2017). However, dendritic orientation in terms of the space-filling growth of the dendritic arbors from the cell bodies in the cerebral cortex has been less explored. Since the early detailed studies of the nervous system (Cajal, 1899–1904), it has been well established that neurons have variable dendritic orientations, ranging from rather asymmetric to symmetric dendritic arbors. It is important to determine the orientation of dendritic arbors in the context of patterns of connectivity: an orientation preference of the dendritic arbors along a given axis would maximize the possibility of an input coming in the same direction making contact with that dendritic arbor. Colonnier (1964) pioneered the study of this question in the cerebral cortex. He was inspired by Young’s studies of the optic lobes of the cephalopod *Octopus vulgaris* performed in tangential sections. Young (1960) found that the shape of the dendritic fields of the neurons of the optic lobe is mostly elongated in the tangential plane, with orientations in mainly two directions at right angles to each other. Young hypothesized that this dendritic orientation could explain the visual discrimination behavior of the cephalopod. Thus, Colonnier examined the basal dendritic trees of visual cortex pyramidal cells in tangential sections in cats, rats and monkeys. He measured this orientation with respect to six angles of 30° and found that the shape of the basal dendritic trees of pyramidal cells is circular or elongated. He also found that the long axes of the elongated dendritic fields may be orientated in any direction, although there is a bias toward the anterior/posterior direction in the cat and rat, and parallel to the lunate sulcus (medio-laterally) in the monkey. Wong (1967) conducted a similar study using similar methods in three areas of the cat auditory cortex (AI, Ep, and association). Wong observed that the shapes of basal dendritic fields ranged from relatively circular to “extremely polar,” although elongated fields were unusual. He concluded that the degree of elongation is greater in the visual than in the auditory cortex.

More recently, Elston and Rosa (1997) examined the orientation of basal dendritic arbors of Layer III pyramidal cells that were labeled using intracellular injections of Lucifer yellow in tangential sections of the monkey cerebral cortex, including: the primary visual area (V1), the second visual area (V2), the middle temporal area (MT), the ventral portion of the lateral intraparietal area (LIPv), and the portion of cytoarchitectonic area 7a within the anterior bank of the superior temporal sulcus. They classified neurons based on polar plots of dendritic branches versus direction from the cell body. Generally, they found that the shape of most basal dendritic fields was circularly symmetrical in the dimension tangential to the cortical layers. However, there were significant biases in orientation (tendency of dendritic branches to form dendritic clusters along particular axes) in certain areas: most neurons in V1 showed a significant bias, most neurons in V2 had some degree of orientation bias, whereas biases were less clear in neurons in the other studied areas. Elston and Rosa (1997) concluded that the fact that a large proportion of cells with narrow morphologically orientation- and direction-biased dendritic fields were found in V1 but not in the other areas could be related to the generation of the orientation selectivity of Layer III by sampling inputs from the underlying Layer IV along given axes of the visuotopic map.

At present, there are a number of methods and software tools to support the study of the local orientation of single cells using polar locations, such as Neurolucida software (MicroBrightField). The Neurolucida polar histogram describes the overall direction of dendritic growth. The growth is displayed in the form of a round directional histogram that uses pie shaped wedges to describe frequencies. Nevertheless, the problem of determining the relative orientation of a group of cells with regard to the same spatial point of reference cannot be solved using these methods. This is because it is hard to retain the spatial localization of the neurons after the samples are processed. Thus, it is only possible to compare the orientation of the dendritic fields of individual cells along particular directions when neurons are located within the same section.

In this paper, we propose a novel method for calculating the orientation of a group of cells according to their physical position in the brain. Furthermore, we estimate local orientation to prevent the loss of dendritic segments in the orientation estimate processing and increase the maximum number of intervals in the polar histogram from 120 (Neurolucida) to 360 used to calculate the orientation of a particular neuron. For this purpose, we use circular techniques to find a match between the orientation of the dendritic arbor of a single neuron and the relative orientation of the neighboring neurons.

We analyzed the dendritic orientation of complete basal arbors of pyramidal neurons in Layers II, III, IV, Va, Vb, and VI of the hindlimb somatosensory (S1HL) neocortex of 14-d-old (P14) rats. We found that their dendrites are not homogeneously distributed around the soma, having instead particular orientations that are not necessarily the same as the neighboring neurons of a particular cortical layer. Furthermore, we did not find any preferred orientation within and between layers the S1HL cortex.

## Materials and Methods

### Data

**Figure 1.**
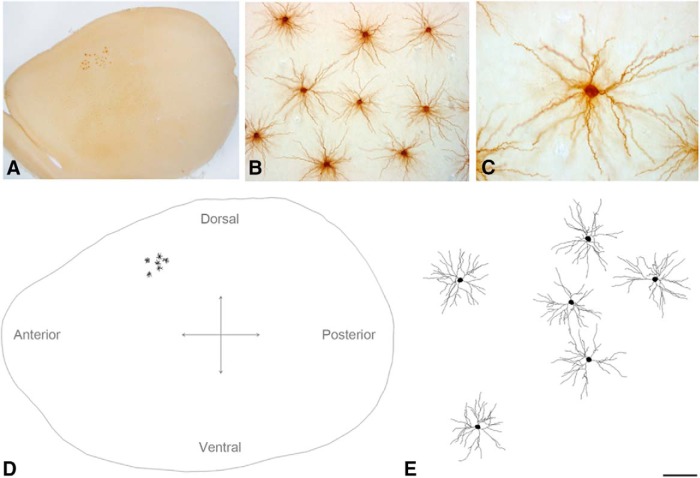
***A***, Low-power photomicrograph showing injected neurons in Layer III from the S1HL cortex, viewed from the plane of the section parallel to the cortical surface of the rat brain. ***B***, ***C***, High-magnification photomicrographs showing examples of pyramidal cell basal dendritic arbors from Layer III. ***D***, Schematic diagram of pyramidal neuron basal dendritic arbors injected in Layer III from the S1HL cortex. ***E***, Higher-magnification image of the neurons shown in ***D***. Scale bar shown in ***E*** indicates 600 μm (***A***), 110 μm (***B***), 50 μm (***C***), 1000 μm (***D***), and 100 μm (***E***).

A set of 288 complete three**-**dimensionally reconstructed pyramidal cell basal arbors from Layers II, III, IV, Va, Vb, and VI of the S1HL neocortex of male Wistar rats (*n* = 20, postnatal day 14, RRID: RGD_5508396) was used for the study (Rojo et al., 2016). The neurons were distributed in 44 horizontal sections (6 ± 2 neurons per brain slice; 150 − 200 μm thick). They are referred to as maps. Each map was composed of the contour of the horizontal section and the real position of each neuron within the section (Fig. 1).

### Circular centralization measures

**Figure 2.**
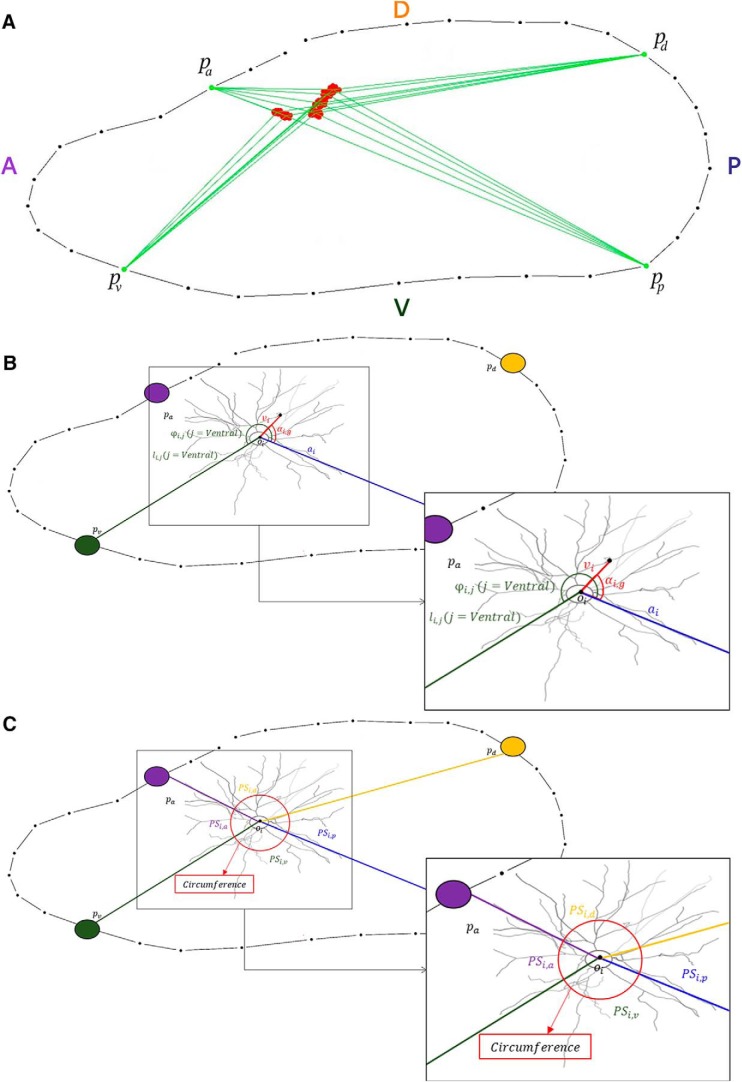
Example of a reconstructed horizontal brain section (map from rat id1II) to describe the orientation determination process. ***A***, Green dots are the arbitrarily placed points $p_{j}$ to indicate orientation direction, where *j* could be *p =* posterior, *a =* anterior, *d =* dorsal, or *v =* ventral. The red dots represent the position of neurons located in the brain. The green lines depict the limit of the direction sector (denoted by colored capital letters) for each neuron. ***B***, This image shows the different angles to be measured for DLRM. The inset shows the angles to be measured: an example of an $\alpha_{i,g}$ angle, where the point *g* is represented as a black dot and an example of a $\varphi_{i,j}$ angle, where *j* represents the orientation (ventral in this example). ***C***, This image shows the proportion of the circumference ${(PS}_{i,j})$ for each orientation sector to be used in the DLRM. The inset shows the red circumference around the center of the neuron $o_{i}$ with the four ${PS}_{i,j}$ sectors corresponding to the four orientations.

We use circular techniques (Mardia and Jupp, 1999; Ley and Verdebout, 2017) to find a match between the orientation of the dendritic arbors of neurons within their physical location in the brain. This measurement is based on the study of the percentage of the dendritic arbor with an arbitrary selected cortical orientation (see below) with respect to the total neuronal dendritic mass (Fig. 2).

Given a set of circular instances${(\theta }_{1},\ldots ,\theta_{n})$, the Fisher median direction (Fisher, 1993) is the value ϕ ϵ ${(\theta }_{1},\ldots ,\theta_{n})$ that minimizes the sum of circular distances:

$$\phi^{*}=\arg\min\left(\pi -\overset{n}{\underset{i=1}{\sum }}\left|\pi -\left|\theta_{i}-\phi \right|\right|\right).$$

Another well-known circular centralization measure is the mean direction (Batschelet, 1981; Mardia and Jupp, 1999; Jammalamadaka and SenGupta, 2001). Given a set of angles ${(\theta }_{1},\ldots ,\theta_{n})$, we get

$$R=\left(\sum_{i=1}^{n}\cos\left(\theta_{i}\right),\sum_{i=1}^{n}\sin\left(\theta_{i}\right)\right)=\left(C_{n},S_{n}\right).$$

The direction of this resultant vector **R** represents the circular mean direction, denoted by $\overset{-}{\theta }$. This is obtained as

$$\overset{¯}{\theta }=\arctan\frac{C_{n}}{S_{n}}.$$

Note that the main disadvantage of the mean direction is the possibility of being affected by outliers.

### Orientation of the neurons in the brain

**Figure 3.**
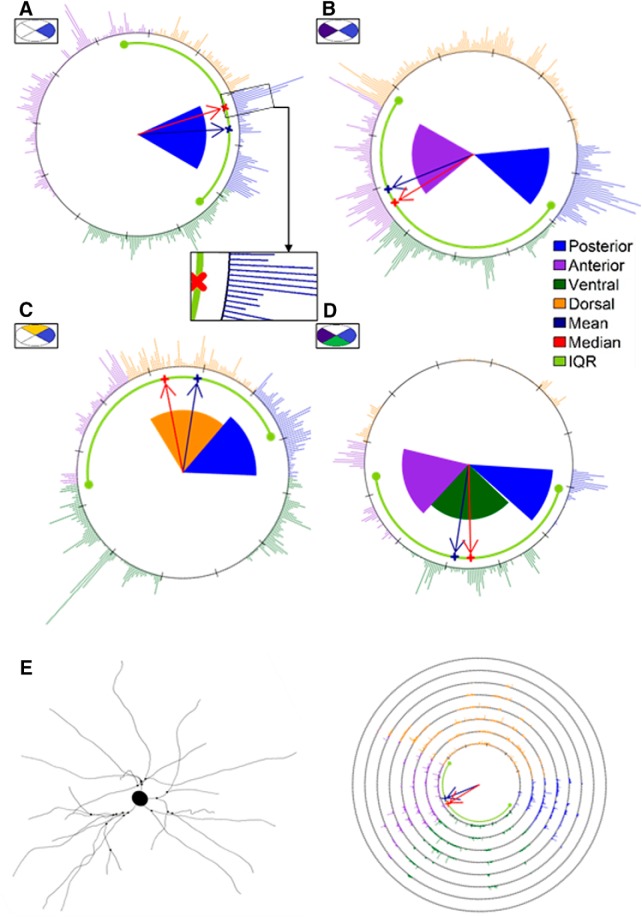
***A–D***, OCH of four example neurons to illustrate the four main orientation types found. The cumulative colored dots represent the neuron dendritic segments of length 0.5μm clustered at each of the 360° around the soma, as shown in the zoomed area of ***A***. Each dot is colored according to its corresponding direction sector. Red and blue arrows indicate the median and mean directions, respectively. Furthermore, a thick light green line represents the IQR, and the big dots located at the ends of the line are the 25th and 75th percentile (first and third quartile). The shaded areas indicate the results of the orientation analysis using DLRM. These are colored according to the color code of the orientation. The darker shades indicate a stronger the orientation in accordance with the 1.1, 1.2, and 1.3 thresholds. The ellipses inside rectangles located above each neuron representation provide visual information about what type of orientation have these neurons. Additionally, colors inside the ellipses should facilitate the visual identification of the four possible orientations: (***A***) single orientation, (***B***) opposite orientation, (***C***) contiguous orientation, and (***D***) triple orientation. ***E***, Neurolucida explorer (left) and ECH (right) representation of the same neuron as in ***B***. Small black dots located in the dendritic arbors in the left-hand image represent the basal dendrite branching points. Each concentric circumference in the ECH representation indicates an additional distance of 25 μm from the soma. The cumulative colored dots located on the circumferences are the neuron dendritic segments of length 0.5μm. Each dot is colored according to its corresponding cortical area growth direction. The remaining OCH and ECH neuron representations may be found in http://cig.fi.upm.es/node/1003.

To objectively establish the orientation of the neurons in the horizontal brain sections, four points of reference $p_{j}$ were placed individually for each map following the typical anatomic orientations (posterior, anterior, dorsal, and ventral) commonly used in brain atlases (Franklin and Paxinos, 1997) indicating *j* = *p* for posterior, *j* = *a* for anterior, *j* = *d* for dorsal, and *j* = *v* for ventral. Every $p_{j}$ was connected by a straight line to the center *o* of each neuron *i* (i.e., to the soma center $o_{i}$ with coordinates $\left(o_{i_{x}},o_{i_{y}}\right)$, with *i =* 1,., maximum number of neurons in the map). This generated four different sectors for each neuron *i*, corresponding to the four orientations (Figs. 2*A*, 3).

The angle $\alpha_{i,g}$ originated by the vector $a_{i}$ with the vector $v_{i}$, where the $a_{i}$ vector components are obtained from $o_{i}$ (the soma center of the neuron i) and $p_{p}$ (the posterior orientation point corresponding to the back of the brain), and the $v_{i}$ vector components are obtained from $o_{i}$ (the soma center of the neuron i) and an arbitrary point of the dendritic wiring from neuron *i* named *g* with coordinates $\left({g_{i}}_{x},{g_{i}}_{y}\right)$, is given by

(1) $$\alpha_{i,g}=\arctan\left(\frac{a_{i_{x}}*v_{i_{y}}-a_{i_{y}}*v_{i_{x}}}{a_{i_{x}}*v_{i_{x}}+a_{i_{y}}*v_{i_{y}}}\right),$$

where $a_{i_{x}}=p_{p_{x}}-o_{i_{x}},a_{i_{y}}=p_{p_{y}}-o_{i_{y}}$, $v_{i_{x}}={g_{i}}_{x}-o_{i_{x}}$ and $v_{i_{y}}={g_{i}}_{y}-o_{i_{y}}$, with $\left(p_{p_{x}},p_{p_{y}}\right)$ being the coordinates of the point $p_{p}$. Any of the four limit points [$p_{j}$ (j = p, a, d, and v)] can be used to calculate this angle (not just $p_{p}$), although we used $p_{p}$ to establish a global reference point for this study.

Given the vector $l_{i,j}$ for the neuron *i* and the limit point *j*, whose components are obtained from $o_{i}$ and $p_{j}$, then the angle $\varphi_{i,j}$ between the vector $a_{i}$ with the vector $l_{i,j}$ is calculated according to Equation 1, where the components of $v_{i}$ are replaced by the components of $l_{i,j}$ with $l_{{i,j}_{x}}={p_{j}}_{x}-o_{i_{x}}$, $l_{{i,j}_{y}}={p_{j}}_{y}-o_{i_{y}}$ and j=p,a,d,v. Note that when j=p, the angle $\varphi_{i,j}=0$.

These $\varphi_{i,j}$ angles are regarded as sectors that represent the four selected orientations of the neuron. Therefore, we were able to associate every $g_{i}$ points of the neuron *i* to a specific orientation *p*, *a*, *d*, or *v*, depending on their $\alpha_{i,g}$ angle (Fig. 2*B*).

We developed two different methods to estimate the orientation of the neurons. These methods are called the Fisher median direction method and the dendritic length ratio method (DLRM).

#### Fisher median direction method

**Figure 4.**
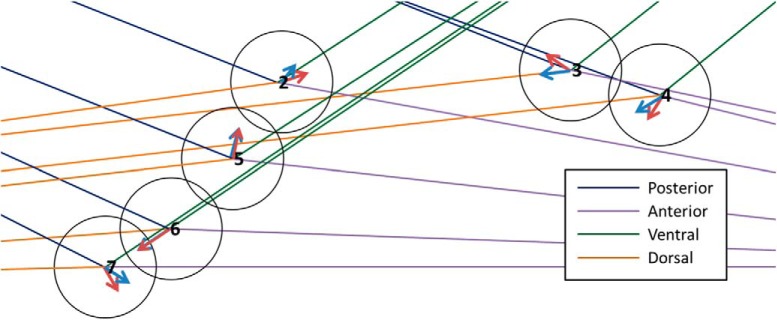
Example of the Fisher median direction orientation method results for a map shown in Figure 2. The numbers are the identification of each neuron from this map. The red arrows are the Fisher median direction for each neuron, the blue arrows are the mean direction for each neuron, and the colored lines represent the orientation sector for each neuron, where the blue line is the posterior direction, the orange line is the dorsal direction, the purple line is the anterior direction, and the green line is the ventral direction. In this example, the orientation of the set of neurons was not consistent, therefore we classified the orientation of this brain map as undetermined.

Since every $g_{i}$ is associated with an angle $\alpha_{i,g}$, as explained above in Orientation of the neurons in the brain, we used the Fisher median direction together with the mean direction, both described above in Circular centralization measures, to determine the orientation of each neuron (Fig. 4). The median direction is considered as the main orientation direction. The mean direction is used for support when the median orientation direction is unclear (i.e., the median direction is oriented toward one of the $p_{j}$ points). Since the orientation of the neurons from the same map is determined according to the same reference point, the orientation tendency of a group of neurons belonging to the same map is determined as the match between the orientations of the neurons individually. This tendency of a group of neurons is determined according to the following criteria:

##### One orientation

• If >60% of the neurons from the same map are oriented towards same *j* orientation, then it is partly oriented towards *j* orientation.
• If >80% of the neurons from the same map are oriented towards same *j* orientation, then it is oriented towards *j* orientation.

##### Two orientations

Note that there must be at least a 30% and a 50% of the neurons from the same map oriented toward two different contiguous orientations respectively to consider them for multi-orientation:

• If >80% of the neurons from the same map are oriented towards two contiguous orientations, then it is partly oriented towards those two contiguous orientations.
• If 100% of the neurons from the same map are oriented towards two contiguous orientations, then it is oriented towards those two contiguous orientations.

This method is simple and easy to apply. Nevertheless, it summarizes the orientation as a single value; hence, there is a considerable loss of information.

#### DLRM

**Figure 5.**
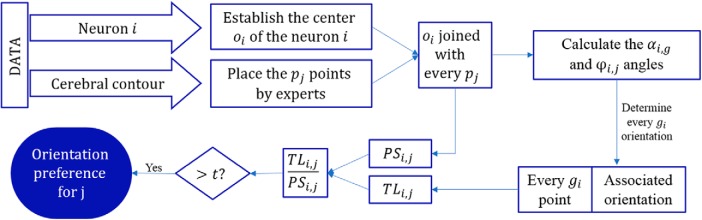
Flowchart representing the algorithm to estimate the orientation of a neuron via DLRM.

The DLRM (Fig. 5) addresses the information loss problem, considering the results provided by every dendritic segment of the neuron.

Since, as explained in detail above in Orientation of the neurons in the brain, every $g_{i}$ point has an associated orientation sector, we estimate the orientation of the neuron *i* as follows: we calculate the proportions of the total circumference of each orientation sector *j* for neuron *i*, which is denoted ${PS}_{i,j}$ (Fig. 2*C*). We also calculate the proportion of the total dendritic length (i.e., the proportion of $g_{i}$ points) that corresponds to each sector *j*, called ${TL}_{i,j}$.

Hence, the growth orientation preference of neuron *i* is determined depending on the values of $\frac{{TL}_{ij}}{PS_{ij}}$:

**Table:** 

If $\frac{{TL}_{ij}}{PS_{ij}}$	>t → Neuron *i* has a growth orientation preference for *j*.
	>1and<t → Neuron *i* has a growth orientation preference for *j*. Nevertheless, $\frac{{TL}_{ij}}{PS_{ij}}$ is not high enough for *j* to be considered as a possible growth orientation preference.
	<1 → Neuron *i* does not have a growth orientation preference for *j*,

where ***t*** is the established threshold for neuron *i* to be considered to have a growth orientation preference for the respective direction. We arbitrarily considered different thresholds (***t*** = 1.1, 1.2, and 1.3) to be able of compare the results in either a constrained scenario such as ***t*** = 1.3 or a less constrained scenario such as ***t*** = 1.1.

We also account for the possibility of a multiple orientation scenario. This means that there is more than one preferred growth direction. The number of possible preferred growth directions for each neuron is from 0 to 3. Therefore, we may find (Fig. 3*A–D*): neurons with a single orientation, neurons with a double, either contiguous or opposite, orientation and neurons with a triple orientation.

The orientation of a group of neurons can be estimated easily as the agreement between the orientations of these neurons individually. This is possible owing to the fact that the orientation of the neurons individually within the same map is always estimated from the same reference point (i.e., the $p_{p}$ point).

### Graphical representation

For the graphical representation of the data from this study, we propose two variants of the well-known circular histogram (Mardia and Jupp, 1999; Pewsey et al., 2013).

The first proposal is called oriented circular histogram (OCH). In this representation, each dendritic segment of length 0.5μm is represented by a dot on the unit grade section of the circumference. We chose 0.5 µm to include as much dendritic information as possible and avoid an overloading of the graph. They are colored according to a color code that identifies the direction in which the segment is oriented. The following shows the color code: orange is the dorsal orientation, dark green is the ventral orientation, blue is the posterior orientation, and purple is the anterior orientation.

For further descriptive information, a red arrow and a blue arrow indicate the median and mean directions, respectively. Furthermore, a thick light green line represents the interquartile range (IQR; Abuzaid et al., 2012), and the big dots located at the ends of the line denote the 25th and 75th percentile (first and third quartile).

Finally, we represented the results of the orientation study for each neuron as a shaded area (Fig. 3*A–D*), colored according to the color code of the orientations. Darker shades denote a stronger the orientation.

Furthermore, we developed another graphical representation, called expansion circular histogram (ECH), which is also a circular plot where we represented the unit grade dots in concentric circumferences (Fig. 3*E*). Each circumference represents the distance to the soma. This provides a visual estimate of the expansion of the neuron. Each circumference denotes an extra Euclidean distance separation of 25μm, e.g., the first circumference contains the dots (i.e., the dendritic segments of length 0.5μm) that are from 0 to 25 μm from the soma, the second circumference includes the points from 25 to 50 μm, the third circumference has the points located from 50 to 75 μm, etc. Furthermore, the median direction within the first circumference is also represented as a red arrow, the mean direction as a blue arrow and the IQR as a thick light green line.

### Software

We used Neurolucida (RRID:SCR_001775) and Neuroexplorer (RRID:SCR_001818) software for data processing.

The R software (R Core Team, 2014; RRID:SCR_001905) was used for data management and to deal with circular statistics (Pewsey et al., 2013). We also implemented the graphical representations for descriptive visualization of the neurons and the algorithms to determine neuron orientation in R software.

### Code accessibility

The code described in the paper is freely available online at https://github.com/ileguey/Patterns-orientation-neuron.git. The code is available as Extended Data 1. These were run in Windows 10 OS.

Extended Data 1 Extended data containing the R code files. Download Extended Data 1, ZIP file.

## Results

We analyzed the dendritic orientation of the complete basal arbors of pyramidal neurons in Layers II, III, IV, Va, Vb, and VI of the S1HL neocortex of P14 rats. To determine the orientation of the neuron, we used the two different methods described in Materials and Methods, Orientation of the neurons in the brain: the Fisher median direction method and the DLRM.

**Figure 6.**
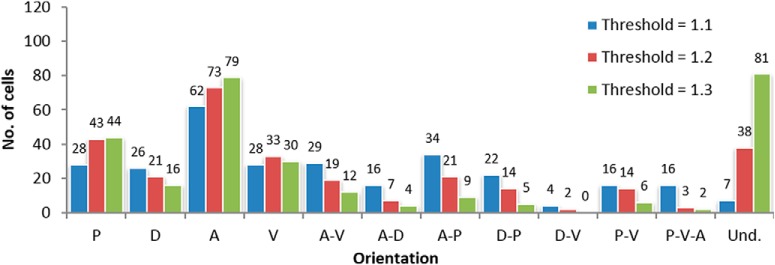
Histogram showing the comparison between the orientation of the neurons in each brain section (*n* = 288) for the different selected DLRM thresholds (blue 1.1, red 1.2, and green 1.3). The frequencies are displayed above each bar.

We found that most neurons had an asymmetric dendritic pattern with four different types of orientations: (1) single orientation, where the dendrites of the neuron are oriented toward a single orientation; (2) contiguous orientation, where the dendrites are oriented toward two orientations that are adjacent to each other; (3) opposite orientation, where the dendrites are oriented toward two orientations that are opposite to each other; and (4) triple orientation, where the neuron is oriented toward three orientations. Furthermore, we found that there is a mixture of the four different types of orientations within any given group of neurons in any cortical layer. Additionally, we investigated whether the dendritic orientation was related to the physical location within the brain with respect to the anterior, dorsal, posterior and ventral directions. The results using the Fisher median direction method showed that the consistency of the orientation of the neurons in 22 out of 44 maps was minimal, and only seven maps could be considered to have an orientation pattern (Table 1, maps 10II, 8III, 14III, 15Va, 3Vb, 12Vb, and 10VI). The results using the DLRM revealed that dendrites do not generally have an orientation preference (Fig. 6). Nevertheless, of the neurons that do have an orientation preference, most are oriented in the anterior direction, almost doubling the number of neurons oriented toward the next preferred growth direction, which is the posterior direction. Furthermore, considering cases of multiple orientation (i.e., there is more than one orientation preference), we found that the anterior direction was again the most common. On the other hand, we observed that the preference for the dorsal direction was by far the least frequent for cases of both orientation and multiple orientation.

**Table 1.** This table shows the information of each brain map analyzed using the Fisher median direction method

Animal ID	Number of cells	P	D	A	V	Map tendency
1 II	6	1	3	0	2	Undetermined
4 II	6	1	2	1	2	Undetermined
5 II	6	0	1	1	4	Partly V
6 II	6	0	2	1	3	Undetermined
7 II	6	3	1	2	0	Undetermined
8 II	6	0	1	2	3	Partly A-V
9 II	6	1	0	1	4	Partly V
10 II	6	0	0	2	4	A-V
1 III	6	0	2	2	2	Undetermined
5 III	6	1	2	2	1	Undetermined
6 III	6	2	1	0	3	Undetermined
7 III	6	0	1	2	3	Partly A-V
8 III	6	0	0	5	1	A
13 III	6	0	3	2	1	Partly D-A
14 III	6	1	0	0	5	V
16 III	6	1	1	2	2	Undetermined
17 IV	15	4	2	3	6	Undetermined
18 IV	17	5	3	5	4	Undetermined
19 IV	11	3	1	4	3	Undetermined
20 IV	5	2	1	2	0	Undetermined
1 Va	6	2	1	1	2	Undetermined
3 Va	6	0	2	3	1	Partly A-D
5 Va	6	0	1	1	4	Partly V
8 Va	6	1	2	3	0	Partly A-D
13 Va	6	2	1	2	1	Undetermined
14 Va	6	1	2	1	2	Undetermined
15 Va	5	0	3	2	0	A-D
16 Va	7	0	3	2	2	Undetermined
3 Vb	6	0	0	0	6	V
4 Vb	6	1	1	3	1	Undetermined
9 Vb	6	3	2	0	1	Partly P-D
10 Vb	6	2	2	1	1	Undetermined
12 Vb	6	1	0	0	5	V
13 Vb	6	1	0	2	3	Partly A-V
14 Vb	6	0	3	1	2	Undetermined
15 Vb	6	1	2	2	1	Undetermined
5 VI	6	2	0	1	3	Partly P-V
6 VI	6	2	3	1	0	Partly P-D
8 VI	6	2	**3**	0	1	Partly P-D
9 VI	6	2	1	1	2	Undetermined
10 VI	6	5	1	0	0	P
11 VI	6	2	0	1	3	Partly P-V
12 VI	6	2	0	1	3	Partly P-V
16 VI	6	1	1	1	3	Undetermined

Animal ID refers to the number of animals from which cells where sampled and the layer in which the cells were sampled (Roman numeral), the number of neurons contained in the map (number of cells), the number of neurons oriented individually toward the posterior direction (P), the dorsal direction (D), the anterior direction (A), and the ventral direction (V). Map tendency represents the orientation of the group of neurons within each map; “undetermined” means that the results cannot be used to decide an orientation for the group of neurons, “partly” means that the results suggest that some of the neurons are specifically oriented.

**Table 2.** This table shows the information of each brain map analyzed according to the DLRM

Animal ID	Number of cells	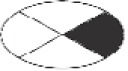 P^S^	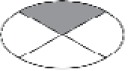 D^S^	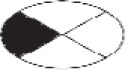 A^S^	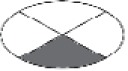 V^S^	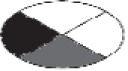 A-V^C^	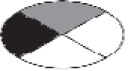 A-D^C^	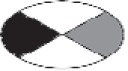 A-P^O^	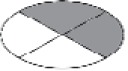 D-P^C^	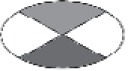 D-V^O^	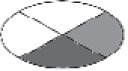 P-V^C^	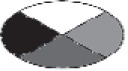 P-V-A^T^	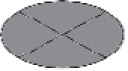 Und.
1 II	6	-	-	1(1)1	2(0)0	1(1)1	-	1(0)0	-	-	-	-	1(4)4
4 II	6	1(2)2	-	1(0)0	2(1)1	-	-	-	1(1)0	-	1(0)0		0(2)3
5 II	6	1(1)1	1(1)0	0(2)3	1(1)0	2(1)0	-	1(0)0	-	-	-	-	0(0)2
6 II	6	0(1)1	-	2(2)1	2(1)0	-	-	-	1(0)0	-	1(1)1	-	0(1)3
7 II	6	2(3)3	1(1)1	1(1)0	1(1)0	-	1(0)0	-	-	-	-	-	0(0)2
8 II	6	-	1(0)0	1(1)1	0(1)1	1(1)0	-	1(1)1	-	-	-	2(1)1	0(1)2
9 II	6	0(2)2	-	1(1)1	-	-	-	3(2)1	-	-	1(1)0	1(0)0	0(0)2
10 II	6	1(1)0	-	5(5)5	-	-	-	-	-	-	-	-	0(0)1
1 III	6	-	-	3(4)4	-	-	-	2(1)0	-	-	-	1(1)1	0(0)1
5 III	6	1(1)2	1(1)0	2(1)1	-	-	1(1)1	1(1)0	-	-	-	-	0(1)2
6 III	6	2(2)1	1(1)1	1(2)3	-	2(1)0	-	-	-	-	-	-	0(0)1
7 III	6	0(1)0	-	2(2)3	-	2(2)1	-	1(0)0	-	-	-	-	1(1)2
8 III	6	-	2(1)0	3(2)2	-	1(0)0	-	-	-	-	-	-	0(3)4
13 III	6	0(1)1	-	0(2)2	1(1)1	-	2(0)0	1(1)0	1(0)0	-	-	1(0)0	0(1)2
14 III	6	-	-	1(1)2	1(1)1	4(4)3	-	-	-	-	-	-	-
16 III	6	-	1(0)0	1(2)2	1(2)0	1(0)0	-	-	-	1(0)0	1(0)0	-	0(2)4
17 IV	15	1(3)3	3(3)2	2(3)3	1(2)1	-	1(0)0	-	4(2)0	1(0)0	1(1)1	1(0)0	0(1)5
18 IV	17	3(3)2	3(3)3	2(3)3	2(2)1	1(1)1	1(0)0	4(3)3	1(1)1	-	-	-	0(1)3
19 IV	11	0(0)2	1(1)1	4(5)5	-	-	1(1)1	3(2)2	2(2)0	-	-	-	-
20 IV	5	2(2)2	-	0(0)1	-	-	-	2(2)1	-	-	1(1)0	-	0(0)1
1 Va	6	-	-	1(1)1	2(2)2	1(1)1	-	1(0)0	-	-	-	-	1(2)2
3 Va	6	-	1(0)0	4(4)4	-	-	-	-	1(0)0	-	-	-	0(2)2
5 Va	6	1(0)2	-	0(1)0	-	1(1)1	-	-	-	-	2(2)0	1(0)0	1(2)3
8 Va	6	-	1(0)0	2(1)3	-	-	1(1)0	1(1)0	1(1)1	-	-	-	0(2)2
13 Va	6	3(3)3	-	1(2)2	-	-	-	1(0)0	-	-	1(1)1	-	-
14 Va	6	0(1)1	2(2)2	2(2)2	-	0(1)0		1(0)0	-	-	-	1(0)0	0(0)1
15 Va	5	1(1)1	2(1)0	0(1)0	-	-	2(1)1	-	-	-	-	-	0(1)3
16 Va	7	-	1(1)1	1(1)1	-	1(0)0	1(0)0	-	2(2)2	-	-	-	1(3)3
3 Vb	6	-	-	2(3)4	1(2)2	2(0)0	-	1(1)0	-	-	-	-	-
4 Vb	6	1(1)2	1(1)0	1(1)1	1(1)1	-	-	1(1)0	1(0)0	-	-	-	0(1)2
9 Vb	6	1(2)4	-	1(1)1	-	-	-	-	4(3)1	-	-	-	-
10 Vb	6	1(1)1	0(1)0	2(1)0	2(2)2	-	1(0)0	-	-	-	-	-	0(1)3
12 Vb	6	-	-	1(1)1	0(1)1	2(2)2	-	-	-	-	1(2)2	2(0)0	-
13 Vb	6	2(2)2	-	-	1(1)1	1(0)0	-	-	-	1(1)0	-	-	1(2)3
14 Vb	6	0(1)0	-	1(1)2	1(0)0	1(1)1	2(2)1	-	-	-	1(0)0	-	0(1)2
15 Vb	6	1(2)1	-	1(3)3	-	2(0)0	1(0)0	1(0)0	-	-	-	-	0(1)2
5 VI	6	0(0)1	-	2(2)2	1(2)3	0(1)0	-	-	-	-	1(1)0	2(0)0	-
6 VI	6	0(2)1	1(1)2	0(0)1	-	-	1(1)0	2(1)0	2(1)0				0(0)2
8 VI	6	1(2)1	2(2)3	-	0(0)1	-	-	1(0)0	1(1)0	-	1(1)0	-	0(0)1
9 VI	6	1(1)0	-	1(1)1	1(2)2	1(1)1	-	1(1)1	-	-	-	1(0)0	0(0)1
10 VI	6	-	-	1(1)1	2(3)4	-	-	-	-	1(1)0	1(1)1	1(0)0	-
11 VI	6	1(1)0	-	2(3)3	-	1(0)0	-	1(1)0	-	-	-	-	1(1)3
12 VI	6	0(0)1	-	1(0)0	1(3)3	1(0)0	-	1(1)0	-	-	2(1)0	-	0(1)2
16 VI	6	0(0)1	-	2(2)3	1(1)2	-	-	1(1)0	-	-	0(1)0	2(1)0	-
Total	**288**	**28(43)44**	**26(21)16**	**62(73)79**	**28(33)30**	**29(19)12**	**16(7)4**	**34(21)9**	**22(14)5**	**4(2)0**	**16(14)6**	**16(3)2**	**7(38)81**

Animal ID refers to the number of the animal from which cells where sampled and the layer in which the cells were sampled (Roman numeral), the number of neurons contained in the map (number of cells), the number of neurons oriented individually toward the posterior direction (P), the dorsal direction (D), the anterior direction (A), the ventral direction (V), the anterior/ventral direction (A-V), the anterior/dorsal direction (A-D), the anterior/posterior direction (A-P), the dorsal/posterior direction (D-P), the dorsal/ventral direction (D-V), the posterior/ventral direction (P-V), the posterior/ventral/anterior direction (P-V-A), and neurons whose orientation is not clearly defined (Und.). The superscript letters indicate the type of orientation (Fig. 3) for the cells contained in the column (S = single orientation, O = opposite orientation, C = contiguous orientation, T = triple orientation). The numbers in the columns from P to Und. determine the number of neurons oriented toward the specific direction, where the first number is the result when *t* = 1.1, the number between brackets is the result when *t* = 1.2, and the third number is the result when *t* = 1.3. The ellipses with shaded areas above the columns represent the type of orientation for the neurons numbered in the respective column, where different gray tones inside the ellipses should facilitate the visual identification of the four possible orientations.

**Figure 7.**
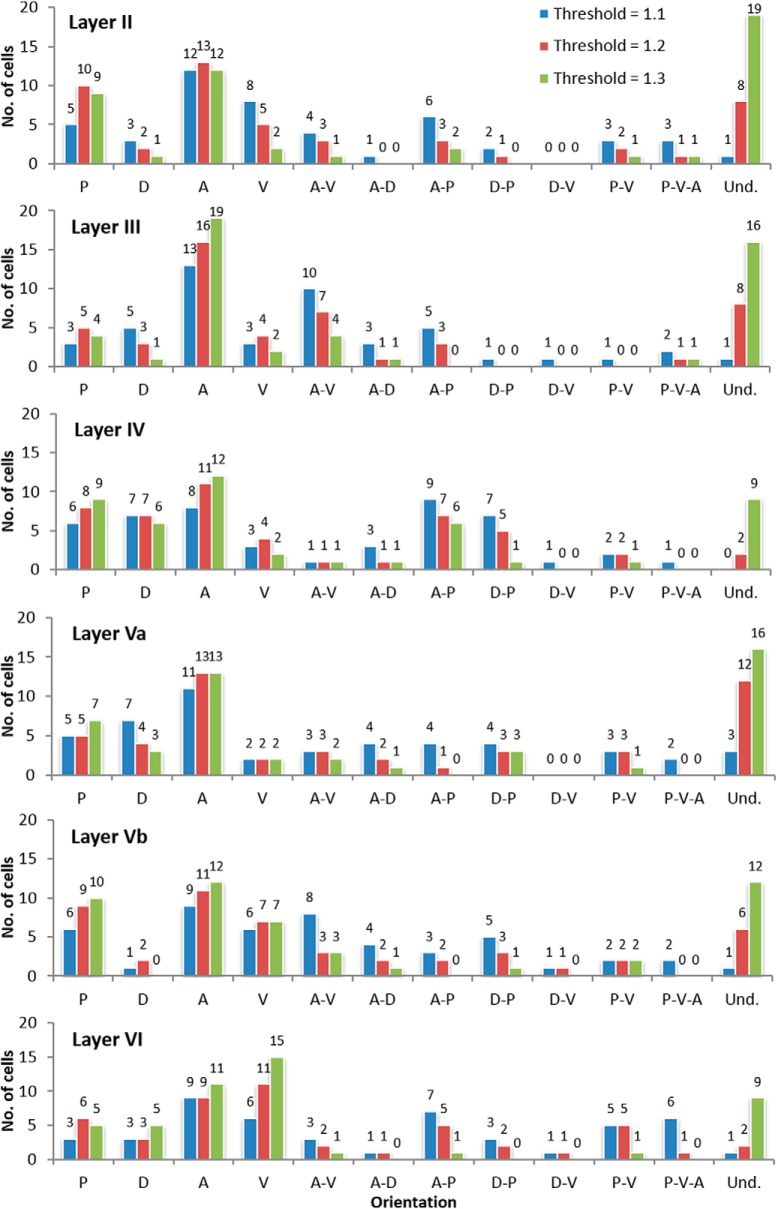
Histogram showing the comparison between the orientation of the neurons sampled from each brain section (*n* = 288) displayed by cortical layers. The different DLRM thresholds (blue 1.1, red 1.2, and green 1.3) are also shown. The frequencies are displayed above each bar.

We then analyzed the orientation of the groups of neurons from each map grouped by layer. The results were quite similar to grouping by orientation (Fig. 7), that is, orientation preference is undetermined. Among neurons with a specific orientation, however, the anterior appeared to be the most frequent and the dorsal, the least frequent orientation. The results are similar, albeit not as clear as when all neurons are grouped together. However, this behavior was not generalized across all layers**:** the anterior as the most frequent applies to neurons from Layers II, III, Va, and Vb, while the dorsal as least frequent orientation applies to neurons from Layers II, III, Vb, and VI. For Layers IV and VI, we were unable to determine the preferred direction among the neurons that have an orientation preference. Nevertheless, we observed that the ventral section is the least preferred orientation for neurons from both Layers IV and Va.

The code average speed was 31,373 s. This was performed in a computer with an Intel Core i7-7700 processor with 3.60 GHz speed and 16.00 GB RAM memory. The total code file size storage is 224 KB.

## Discussion

In this study, we presented two proposals for studying the orientation of dendritic arbors: a method to estimate the orientation of either a single neuron or a neuron group and two novel graphical neuron representations. We first used a method based on the Fisher median direction and the circular mean direction. The results were not accurate enough as this method determined the orientation for only one value. Hence, we proposed another method that complements the Fisher median direction by taking into account more than one value to determine the orientation of a neuron and avoiding the huge loss of information along the process. This method is based on the ratio between the expected and the observed dendritic length, called DLRM. Furthermore, we are able to analyze data in higher detail than Neurolucida, since we use up to 360 intervals instead of 120 that Neurolucida allows. In this study, we also proposed two graphical descriptive representations of the neuron, both based on circular histograms (Mardia and Jupp, 1999; Pewsey et al., 2013). The first representation is called OCH, illustrating the orientation results provided by the Fisher median direction method and the DLRM applied to a single neuron. This graphical representation also provides an overview of the amount of dendritic mass around the soma. The second graphical representation proposal is called ECH. ECH differs from OCH mainly in one aspect: ECH provides detailed visual information about the neuron dendritic expansion rather than the neuron orientation, where several concentric circumferences highlight the amount of dendritic mass at a specified distance from the soma. These two graphs provide a better comprehension of the dendritic organization of neurons and an overview of the individual results provided by the methods proposed in this paper. Using these circular statistics and graphical descriptive representations of the neuron, the main finding is that the dendritic basal arbors of the vast majority of pyramidal neurons from the HL somatosensory cortex of the P14 rats are not distributed homogeneously around the soma but have a preferred orientation. Specifically, we found four different types of orientations: single orientation, where the neuron is oriented toward a single orientation; contiguous orientation, where the neuron is oriented toward two adjacent orientations; opposite orientation, where the neuron is oriented toward two orientations located opposite each other; and triple orientation, where the neuron is oriented toward three orientations. Furthermore, we found that there is a mixture of the four different types of orientations within any given group of neurons in any cortical layer. Nevertheless, the orientation preference was generally toward the anterior orientation, whereas the dorsal orientation is the least preferred orientation. Indeed, ∼45% out of the total set of neurons had an orientation preference toward the anterior orientation, whereas only ∼10% out of the total appear to be oriented toward the dorsal orientation. The comparison across layers revealed that the orientation preference for the anterior orientation was more pronounced in neurons located in Layers II, III, Va, and Vb than for the neurons located in Layers IV and VI. The dorsal orientation was the least preferred orientation in all layers, except for IV and Va where the ventral orientation was the least preferred orientation.

We used rats sacrificed on postnatal day 14, and previous studies (Petit et al., 1988; Kasper et al., 1994) have shown that the dendritic morphology of pyramidal neurons changes greatly during the next postnatal days up to 60 d. Furthermore, these changes occur differently in superficial and deep layers since pyramidal neurons are generated in successive waves during development following the characteristic inside-out pattern of the mammalian neocortex (Sidman and Rakic, 1973; for recent studies, see Englund et al., 2005; Cooper, 2008). Thus, the differences observed between the superficial and deep layers may have to do with the differential degree of maturation of the different layers at P14. Additionally, the orientation preference of the dendritic arbors found in this study might not be the same in the adult neocortex as a consequence of cortical maturation. Further studies using the same methods in the adult neocortex would be required to find out whether orientation preferences change from P14 to adult.

There are several studies indicating that the morphology of the basal dendritic arborization of the pyramidal neuron, such as number of dendrites, dendritic length, dendritic thickness, as well as the number of nodes and endings of dendrites, strongly contributes to their functional diversification (Papouts et al., 2017, and references contained therein). However, other than research related to modeling the generation of orientation selectivity in visual cortex cells, relatively few studies on dendritic orientation deal with the possible functional significance of the different patterns of dendritic arborization (Ledda and Paratcha, 2017). In the visual cortex, for example, Jia et al. (2010) addressed whether or not the preferential firing of neurons in the visual cortex in response to a moving visual stimulus of a particular orientation is related to a hypothetical clustering of orientation-specific synaptic inputs around single dendrites. Jia et al. (2010) used a novel *in vivo* imaging technique to measure input signals in the mouse visual cortex and found that inputs that share the same orientation preference were widely distributed across various dendritic branches of a single neuron, rather than being clustered in the same dendrite. Jian et al. concluded that, instead of single dendrites functioning as discrete computational units, the whole dendritic arbor constitutes the spatially distributed synaptic inputs used to compute the characteristic output firing in response to particular stimulus orientations.

Along the same lines, it has recently been shown that the circularity and uniformity of dendritic arbors in the cat visual cortex is independent of the somatic position in the orientation map (Levy et al., 2014; see also Wilson et al., 2016). The non-correlation between dendritic geometry and the organization of orientation maps is consistent with a study by Martin and Whitteridge (1984) who reported that asymmetric dendritic trees do not predict the neuronal preference for stimulus orientation. Of course, this does not preclude the idea that the geometry of the dendrites is not related to other particular functional attributes. Certainly, the orientation of the dendritic arborization is related to the map of synaptic connectivity of the neurons, and therefore their geometrical features are related to the inputs that they receive. Indeed, the dendritic arborizations of pyramidal cells are generally thought to be specifically oriented to make contact with relevant synaptic inputs in the apical and basal dendrites. Also, activity-independent cues are thought to possibly dominate during the embryonic period, whereas activity-dependent mechanisms might be more relevant during synaptogenesis that occurs postnatally (for review, see Wong and Ghosh, 2002). The fact that we observed that there is a mixture of the four different types of basal dendritic tree orientations in neighboring neurons in any one cortical layer indicates that these geometries are unrelated to any local connectivity “characteristic” possibly existing in that particular layer. Thus, the different orientations may be related to synaptic competition mechanisms possibly occurring during synapse establishment with the same locally available set of axons. Nevertheless, it is striking that a large proportion of cells show a preference for the anterior orientation axis, suggesting that the basal dendrites of pyramidal cells of the rat hindlimb possibly have a preference for an axonal system oriented in this direction.

Finally, it is well documented that pyramidal cell structure varies not only between different cortical areas and species but also in different cortical layers (Larkman, 1991; Elston and Rosa, 1997; Elston et al., 2001; Jacobs et al., 2001; Elston and Rockland, 2002; Ballesteros-Yáñez et al., 2010; Bielza et al., 2014; van Aerde and Feldmeyer, 2015; Leguey et al., 2016; Rojo et al., 2016; for review, see Elston, 2003; Elston et al., 2011). However, relatively few detailed studies have been conducted on dendritic orientation regarding particular axes. Therefore, further studies are required to find out the extent to which the orientation of dendritic arbors differs between different cortical areas or to define the common patterns of dendritic growth in all cortical areas, cortical layers, and species.

## Synthesis

Reviewing Editor: Alfonso Represa, INSERM Decisions are customarily a result of the Reviewing Editor and the peer reviewers coming together and discussing their recommendations until a consensus is reached. When revisions are invited, a fact-based synthesis statement explaining their decision and outlining what is needed to prepare a revision will be listed below. The following reviewer(s) agreed to reveal their identity: Anca Doloc-Mihu, Hongwei Dong.

Dear authors,

With both referees we appreciate your manuscript, and agree with the interpretation of your data that provide advance in the field by helping on the characterization of dendritic features and how these features contributed to distinctive neuronal cell types. We also appreciate the hypothesis that dendritic arborizations of cells are oriented towards making contact with relevant synaptic inputs.

There are number of comments and points that we would like to be revised and that I'm listing below.

Major issues:

- There is a debate on using median over mean. Can you comment on this issue? Which one of these measures seems to be better fitted to your results?

- How many orientation sectors are per neuron? How to determine (algorithm) an orientation sector? Precisely, why using 0.5 um as a threshold? Why not 0.1 or 0.15? How was this threshold found/picked?

- Is your software freely available (scripts)? Where reader can download it from? Concerning Neurolucida and Neuroexplorer, we think that you should make your code/scripts available for reader and for review.

- Why using the two methods for finding the orientation? Can we compare them against each other? That is Fisher median vs DLRM results in a table/figure, pointing out which one is best out the two.

- You must provide clear criteria for classifying onto undetermined, partly groups.

- In Discussion you say “we proposed another method that outperformed the Fisher median direction”. I do not see results that prove this statement.

- Why picked 4 regions: posterior, anterior, dorsal and ventral? Why not 6 or 8? How did you chose them? Please explain in detail. Did you see any advantage of having 4 regions over 6 or 8? Wouldn't more regions provide more accuracy for the results?

- Errors on Table 1, where similar neurons were classified differently. Please explain.

- I think you can go deeper into the Discussion. For eg, Case 17IV14 in OCH and ECH are two different visuals, but you do not explain why even for 0.5um. Is it better to look at ECH since it provides more details?

- Most cases have 6 cells. Please comment.

- Intuitively, more cells more information. So more accurate results from cases 17,18,19 IV. Suggest having more cases showing more than 15 cells.

- Suggest adding a Definitions section where you define clearly what anterior direction, orientation, region mean, for eg. Symmetric and Asymmetric dendritic arbors, polar histogram, orientation sector, ETC.

- Is 45% enough to setup a trend? Usually is above 80% to be believable. Please comment.

- On supplemental figures: for eg, case 17IV14 OCH method - legend does not explain the significance of pink/light purple colors. You must include a shades legend to explain the data correctly.

Minor issues along with suggestions to improve clarity of paper:

Abstract:

- “cortex of 14-day-old rats ” (P14) add for clarification of notation

Significance Statement:

- typo: these cells

Introduction:

- “asymmetric to symmetric dendritic arbors”. You need to define these terms.

- “we more accurately and flexibly estimate local orientation” - This statement is not proven by results. Precisely, what is the accuracy measure and its results? I suggest you add such a measure and the results showing that your method outperforms (via numbers, figure, table).

- “polar histogram” is never defined in paper

Materials and Methods:

- 3.2 “We use circular techniques” - need to add references

- “ with an arbitrary selected cortical orientation (anterior, posterior, ventral and dorsal)” Why not 6 or 8 such orientation regions? What is the angle between these? Are they the same sizes? Where does one (for eg, anterior) start and stop?

- Fisher median direction formula: Incorrect formula: argmin of pi is known, right? So parenthesis before pi and after the sum are missing to make sense on this calculation. Also argmin is calculated over phi? What is the measurement done with these |.| ?

- 3.3 maybe objectively is a better word to use instead of arbitrarily

- “four points were placed on each map indicating: (j = p (posterior), a (anterior), d (dorsal) and v (ventral). Points j were” :

- How were they placed? What was the criterion? Why 4 and not less or more?

- reformulate it for clarity: four points pj were ... indicating j=p for posterior, ...

- plural is confusing. use singular for clarity. you want to say that each point connects to each neuron, righ?

- The writing of the whole paragraph of Eq 1 “Given ...pp” is very difficult to follow. Make it easy to read: The angle between ... is given by..., where ... and put all the details.

- Also, why use Pp when you can use everywhere Pj as defined above.

- Please explain why the Figure 3b shows an angle between 2 points from different groups (eg, posterior and ventral). Does formula work with both points being within the same group? Why or why not? Explain.

- Para “Given the vector ...=0” offers a nice explanation. You use here j = p,d,a,v Why not use this notation everywhere instead of pp?

- 3.3.1 - “Since the orientation of the neurons from the same map is determined according to the same reference point, the orientation tendency of a group of neurons belonging to the same map is determined as the match between the orientations of the neurons individually. ” This paragraph repeats at the end of section 3.3.2. Remove it from either section, and re-write it.

- 3.3.2 - Add a Figure showing this algorithm. Show all 3 cases, TLij and PSij.

- “We considered different thresholds (t = 1.1, 1.2 and 1.3- )“ How did you get these thresholds? Please explain. Why these values?”

- 3.4 - “the third circumference has the points located from 50 to 75က03;ဇE4;ဇ49;, etc.” Up to how much? On average, what was the size? What was the size of the longest of all neurons?

- Would like to see one example figure that includes all means and all medians for all these circumferences. How would they look like? Are these sparse or clustered? Does it show that median is better to use than mean?

- 3.5 - Where are these scripts available?

Results:

- “the Fisher median direction method and the DLRM” - Why? Is that you wanted to compare these two methods to see which one gives more accurate results? Can we see these results?

- “towards three regions” - contiguous or not? Is there any result regarding these 3 contiguous regions?

- “only seven maps could be considered to have an orientation pattern” - You must point reader to these maps.

- “the anterior appeared to be the most frequent” - seen this in all layers except VI

- “the dorsal, the least frequent orientation area.” - This is correct for layers II, Vb and VI. For layer III, D have same as V. And for layers IV and Va D shows more than V. So this statement is not correct (50% -50%, not quite enough to asses). Or am I missing something. Please explain.

- “Nevertheless, we observed that the ventral section is the least preferred orientation for neurons from both layers IV and Va.” - Data shows same numbers in layer IV as in layer III? Explain.

-We assume that reconstructions of neurons were performed in 2D with Neurolucida. Can the authors discuss whether their conclusion would be different if they are able to observe and analyze dendritic orientations in 3D?

Discussion:

- Once you add the suggested figures and results, discussion can go deeper on these results.

References:

- Englund,et al - Replace ... with real names

- Jacobs, et al - Replace ... with real names

- Rojo et al appears twice; 1st ref is incomplete

Figures:

- Figure 1; it might be nice to provide raw images of intracellularly labeled pyramidal neurons.

- Figure 2 illustrate four possible orientations of the basal dendrites. It might be nice to show graphic reconstructions of representative neurons with basal dendrites in those 4 orientations. In anycase the figure needs improvement:

legends are too small, especially the colors

each circle should be made bigger, both of them should take the whole page width

lines need to be ticker, they are not readable

DLRM - expand term

Provide simplified visual information

“different gray tones” - Is there a reason for using gray tones? Please explain. In my opinion you should use same colors as shown.

- the ellipses - Move ellipses to empty spaces, to have more space for big circles.

- (B) opposite orientation - Error: ellipse shows a symmetrical opposite orientation, which is obviously not the case in Figure 2 B. Explain.

- ECH , OCH - expand terms

- “Black dots” - I see only one dot in Fig 2E. What I am missing? Please explain.

- Figure 3 needs improvement:

- “ The blue dot is the center of the map.” The dot is barely visible. Initially, I couldn't see it. However, what is its significance? How is this dot obtained? Please explain.

- Figure 4 needs improvement:

“ The red arrows” lines are too thin to be visible, they look like pixels not continuous lines; make them thicker. also arrows are not visible - make them ticker; add symbols

colors are almost not visible

- Table 1:

- Why the two methods do not have the same groupings; P,D,A, V, AV, AD, AP, DP, DV, PV, PVA, undetermined?Explain.

- What is the criterion for declaring them undetermined? What is the threshold for being “partly”? - -You must give clear criteria for separating the neurons within these groups.

- Why is 1III classified as undetermined and not as triplet? Please explain

- Why is 5Va classified as undetermined and not as partly? Please explain. It shows same data as 5II.

- Table 2: - “The superscript letters” - they are too small; figure caption should not run a new page; Why there is no s or t in the results? Please explain.
